# Effectiveness of psychological support in patients undergoing primary total hip or knee arthroplasty: a controlled cohort study

**DOI:** 10.1007/s10195-015-0368-5

**Published:** 2015-07-29

**Authors:** V. Tristaino, F. Lantieri, S. Tornago, M. Gramazio, E. Carriere, A. Camera

**Affiliations:** Department of Prosthetic Surgery, Santa Corona Hospital, ASL 2 - Savonese, via XXV Aprile 38, 17027 Pietra Ligure, SV Italy; Health Science Department, Biostatistics Unit, University of Genoa, via Pastore 1, Genoa, 16132 Italy

**Keywords:** Psychological support, Hip arthroplasty, Knee arthroplasty, SF-36, Hospital anxiety, Depression scale

## Abstract

**Background:**

We hypothesised that psychological support would have a significant improvement on the mental and physical recovery of patients undergoing primary total hip or knee arthroplasty.

**Materials and methods:**

200 patients were consecutively alternately assigned (1:1) to receive routine care (control group) or, in addition, psychological support from a professional psychologist (experimental group). The psychological support was provided at the pre-operative visit, during the hospitalisation period and at the rehabilitation centre.

**Results:**

Upon discharge, based on the ‘Hospital Anxiety and Depression Scale, a state of anxiety was observed in 12.8 % and 78.9 % of the patients in the experimental and in the control group, respectively (*p* < 0.0001). A state of depression was observed in 12.8 % and 73.7 % of the patients in the experimental and in the control group, respectively (*p* < 0.0001). With regard to the ‘Physical Component Scale’ of the SF-36 questionnaire, a similar temporal trend of values was observed in the two study groups, significantly increasing over time in both groups, taking into consideration both the joint population and the two hip and knee populations separately (*p* < 0.0001). With regard to the ‘Mental Component Scale’ of the SF-36 questionnaire, in both the joint population and the two hip and knee populations separately, an exact opposite temporal trend was observed in the experimental group compared to the control group (*p* < 0.0001), with generally higher scores in the experimental group (*p* < 0.0001). In patients with hip arthroplasty, the average time to reach the physiotherapy objective (i.e., the patient ability to walk 50 metres independently and to climb 10 steps) was 6.7 ± 1.8 days (range 4–12) in the experimental group and 7.9 ± 2.2 days (range 0–13) in the control group (*p* = 0.0015).

**Conclusions:**

In summary, there was a lower incidence of anxiety and depression and better mental well-being in the group of patients who received the psychological support. Within the hip arthroplasty group, the patients who received the psychological support reached the physiotherapy objective 1.2 days earlier than the patients in the control group (*p* = 0.0015).

**Level of evidence:**

Level 3, Non-randomized prospective controlled cohort.

## Introduction

Primary total hip arthroplasty and primary total knee arthroplasty are established elective operations to resolve most severe arthritic conditions affecting the two major lower limb joints. They are two highly successful orthopaedic interventions in terms of overall functional recovery for the patient and the incidence of complications. In spite of this, however, the journey that the patient must take is not without difficulties in terms of the emotions that he or she may experience in the months leading up to the operation, during the stay in hospital, during rehabilitation and in the first few months after the operation.

A patient who makes the choice to have a hip or knee arthroplasty operation experiences periods of anxiety and depression, as already reported in many recent studies. Anxiety and depression are emotions that are already present in the period before the operation [1] and impact on the post-operative progress [2–6]; however, generally speaking, the satisfaction that results from these two types of operation can be considered as undisputed [7].

In the short term, a patient’s recovery of functionality after the operation is mainly linked to clinical factors, e.g., the extent of the surgical trauma, but in the long term it is more closely linked to the degree of functionality before the operation and the patient’s emotional [8] and psychological reaction (anxiety) to the operation [9]. The patient’s reaction is not just understood as his or her physiological response to the operation from a physical point of view, but it also comprises a component that is already partly present in the periods prior to admission combined with an element of the patient’s psychological disposition. Practical implications concern the contemplation of psychological factors and the treatment of psychological symptoms in rehabilitation [10] and the person’s social and functional readjustment [11–13].

Therefore, it seems logical to evaluate whether psychological support therapy which accompanies patients from their admission to hospital until their discharge can impact on the surgical outcome during the rehabilitation period and in the first few months following the operation.

Although various controlled clinical studies have already documented the effect of psychological support in patients who have undergone cardiovascular surgery [14], the removal of breast cancer [15] and gastric band surgery [16], we are not aware of any controlled studies relating to patients undergoing hip or knee arthroplasty operations.

It is for this reason that this controlled cohort study was planned, with the aim of determining the effectiveness of psychological support in patients undergoing primary total hip or knee arthroplasty. We hypothesised that psychological support from a professional psychologist would significantly improve the mental and physical recovery of patients. The patients with and without psychological support therapy were examined by means of standard questionnaires completed by the patient (‘patient reported outcome measures’) and by measuring rehabilitation time.

## Materials and methods

Between February 2011 and May 2012, 200 consecutive patients on a waiting list for an elective operation for primary total hip or knee arthroplasty at the Department of Prosthetic Surgery of Santa Corona Hospital (Pietra Ligure, SV, Italy) were enrolled in the study. To be eligible they had to meet the following inclusion criteria—(1) first prosthetic hip or knee replacement; (2) no psychiatric history at the time of enrolment; (3) no degenerative nervous system diseases; (4) aged <80 years; (5) initial decision to carry out rehabilitation at the physiotherapy centre was referred through the hospital; and (6) provided informed consent for participation in the study and processing of personal data.

Each patient who met the inclusion criteria was consecutively alternately assigned to one of two groups (1:1), with the allocation of the first patient chosen at random by tossing a coin, before the operation. The experimental group (EXP) consisted of patients who, in addition to routine treatment, received psychological support from a professional psychologist and the control group (CTR) consisted of patients who only received routine treatment.

The surgical team was blinded to the treatment arm.

After enrolment, the patients who had experienced intra- or post-operative complications or for whom more than one item of data was missing were excluded (Table 1). Patient demographics are documented in Table 2.

**Table 1:** Study population

	EXP		CTR	
	Hip arthroplasty group	Knee arthroplasty group	Hip arthroplasty group	Knee arthroplasty group
No. of patients in the initial cohort	63	37	66	34
No. of patients excluded from the study (reasons for exclusion)	2 (>1 data item missing)	4 (2 had >1 data item missing; 2 had post-operative complications)	3 (2 had >1 data item missing; 1 had post-operative complications)	2 (1 had >1 data item missing; 1 had post-operative complications)
No. of patients with pre-op SF-36 available	61	33	63	32
No. of patients with HADS available	61	33	63	32
No. of patients with physiotherapy assessment available	59	33	61	32
No. of patients with SF-36 at 45 days available	61	33	63	32
No. of patients with SF-36 at 4 months available	60	33	63	31

**Table 2:** Patient demographics

	All patients		Hip arthroplasty group		Knee arthroplasty group	
	EXP	CTR	EXP	CTR	EXP	CTR
No. of patients	94	95	61	63	33	32
Age at surgery (mean ± SD; years)	61.4 ± 8.7	64.5 ± 8.1	59.9 ± 8.4	63.7 ± 8.7	64.2 ± 8.6	66.1 ± 6.6
Gender (M/F)	45/49	56/39	36/25	31/32	13/20	8/24

### Routine treatment

As normal practice at our institution, the surgeon during the pre-operative meeting with the patient provided him/her with operation-related information, as well as using a standard information brochure as a guide. The information explained (1) what arthroplasty is and why arthroplasty is performed, (2) what a prosthesis is, (3) what type of prosthesis is chosen, (4) the surgical planning, (5) some information on the surgery itself, and (6) what to do after discharge (i.e., physical exercises, lifestyle, clinical follow-up visits). The pre-operative meeting between the surgeon and the patient took place before patient allocation to one of the two arms.

### Psychological support

The psychological support was provided by a professional psychologist (author VT) and focused on the type of clinical procedure within the scope of hospital health psychology. The activity was carried out over the course of four sessions between the psychologist and the patient, lasting about half an hour each time. One session was carried out in the pre-operative period, two during the hospital stay and one during the stay at the rehabilitation centre (Table 3). The protocol for the psychological support activity was developed by the psychologist after 1 year of non-participant observation at the Department of Prosthetic Surgery, aimed at defining the psychological themes and concepts on which to focus the activity. The protocol can be summarised as follows:

**Table 3:** Study synopsis

Time period\activity	Pre-operation	Hospital stay	Rehabilitation centre stay	Upon patient discharge	45 days after surgery	4 months after surgery
Psychological support	EXP (1 session)	EXP (2 sessions)	EXP (1 session)	–	–	–
HADS compiling	–	–	–	EXP	–	–
				CTR		
SF-36 compiling	EXP°	–	–	–	EXP	EXP
	CTR				CTR	CTR
Physiotherapic assessment	–	–	EXP (each day)	–	–	–
			CTR (each day)			

° Following the first session with the psychologist

1. Ascertainment of correct comprehension of the medical and supporting information and clarification of any doubts and misunderstandings (at the time of admission). It must be noted that, from the perspective of health psychology [17], the provision of health information about the risk factors corresponds to an increase in the level of information with a possible increase in anxiety and consequent use of dysfunctional strategies. For this reason, it is now increasingly common to find the term ‘psychoeducational’ associated with health care programmes, including in the specific field of arthroplasty [18, 19].
2. The patient’s personal history and discussion of the psychological experiences linked with the illness and the prescription/decision to undergo an arthroplasty operation (at the time of admission).
3. Processing the emotional states associated with the operation and support to manage them (at the time of admission and during the stay in hospital).
4. Modulation of stress and emotional and behavioural reactions associated with the recovery. Reinforcement of the awareness of perceived self-efficiency associated with the results in the short, medium and long term by explaining to the patient their active role in the healing process (during the stay in hospital and in the rehabilitation centre).
5. Discussion with the patient regarding his/her discharge from hospital, returning home and the check-up visit schedule (during the stay at the rehabilitation centre).

The various phases followed on from one another in a way which was personalised to each patient’s psychological needs and shaped gradually to tackle the various phases (from admission to rehabilitation). During all of the phases, the psychologist also used as a guide the standard information brochure that was already provided to the patient by the surgeon during the pre-operative meeting.

### Patient evaluation (evaluation programme in Table 3)

#### Patient questionnaires

The ‘Hospital Anxiety and Depression Scale’ (HADS) questionnaire [20] was completed by patients from both groups at the end of the hospital stay. The HADS is a widely used questionnaire consisting of 14 items which comprise 2 scales—7 items relating to the scale to measure anxiety (HADS-A) and 7 items relating to the scale to measure depression (HADS-D). Each item is given a score between 0 and 3, so the total score for each scale ranges from 0−21. Values between 0 and 7 indicate a ‘normal’ state of the patient, while higher values indicate a degree of anxiety and depression starting from ‘mild’ (8–10), then ‘moderate’ (11–14), and lastly ‘severe’ (15–21).

This questionnaire is useful to evaluate problems of anxiety and depression in hospitalised patients and patients affected by any physical disease which forces them to undergo medical treatment. The grading of the two variables—anxiety and depression—in this specific study should not be incorporated in a clinical-pathological perspective, but in a perspective that considers anxiety and depression as physiological components of the contingent situation experienced by the patient.

The SF-36 questionnaire was completed by patients from both study groups during the pre-operative visit (the same day as admission but after the first session with the psychologist), at the follow-up on day 45 and at the 4-month follow-up after surgery. The questionnaire consists of 36 questions with multiple-choice answers which make up 8 sub-scales—‘physical functioning’, ‘role-physical’, ‘bodily pain’, ‘general health’, ‘vitality’, ‘social functioning’, ‘role-emotional’ and ‘mental health’. Each scale is converted into a scale ranging from 0−100, with the assumption that each question carries the same weight in the final total. The lower the score is, the worse the impairment and vice versa (i.e., 0 indicates the maximum impairment, while 100 indicates no impairment). It is possible to obtain two indices from these 8 sub-scales—the ‘Physical Component Summary’ (PSC) index, comprised of the first four sub-scales listed above and the ‘Mental Component Summary’ (MCS) index, comprised of the last four sub-scales. These indices represent two mathematical calculations which allow us to establish how important the physical and mental components are in the patient to determine their state of well-being [21].

#### Physiotherapy sheet

During the stay at the rehabilitation centre (8 days following the 5 post-operative days spent in hospital), the physiotherapy evaluation sheet was filled in daily for each patient as routine practice. The information on this sheet regarding the time taken between the start of physiotherapy at the rehabilitation centre and reaching the physiotherapy objective, defined as the ability to walk 50 metres independently and to climb 10 steps (i.e., objective defined as the potential minimum for discharge), was analysed for this study. This parameter was defined in this study as ‘delta autonomy days’. The physiotherapist was blinded to the treatment arm the patient was assigned to.

### Data analyses

The following were analysed:

- The presence of anxiety and depression using the HADS questionnaire. The results of each of the two scales (anxiety and depression) were divided into two categories—no anxiety or depression (values between 0 and 7) and presence of anxiety or depression (values between 8 and 21). The comparison between the experimental group and the control group was made in the joint population and in the two separate populations of patients with hip arthroplasty (referred to here as the ‘hip population’) and the patients with knee arthroplasty (referred to here as the ‘knee population’). The groups were compared using the chi-squared test with Yate’s correction or by Fisher’s exact test where more feasible.
- The scores relating to the SF-36 questionnaire, collected at various time intervals (pre-operative, on day 45 after the operation and at 4 months after the operation). At each follow-up, a comparison was made between the groups using the student’s *t* test for independent samples. Considering the relatively low number of samples, the type of data and their increased variability, especially in the sub-scales, the Mann–Whitney nonparametric test was also applied, which fully confirmed the statistical results of the t-test. The temporal trend of the PCS and MCS scales and of all the sub-scales making up the SF-36 score was analysed in the experimental group and control group by means of a two-way repeated measures analysis of variance (ANOVA). This analysis simultaneously compares the difference between the samples and between the detection times and highlights any behavioural differences (interaction) between the groups. The comparison of the results was made in the joint population and separately within the ‘hip population’ and the ‘knee population’.
- The ‘delta autonomy days’, separately within the ‘hip population’ and the ‘knee population’. The analysis was carried out using the Student’s *t* test for independent samples and the results were confirmed through the Mann–Whitney test.

Considering the type and the distribution of the data and given the accordingly similarity of the statistical results obtained with the parametric test and with the nonparametric test, the data relating to the eight sub-scales, the two SF-36 score indices and the physiotherapy evaluation were summarised as an average and standard deviation and the *p*-values reported refer to the parametric test.

For all of the comparisons between the groups, a *p*-value of <0.05 was considered to be significant. The statistical analysis was carried out with the SPSS 17.0 software.

The data were collated by the first author (VT) and analysed by a statistician (a co-author; FL). The statistician was blinded to the treatment arm and to what the numerical measures meant.

### Sample size determination

This is an original research study in the field of hip and knee arthroplasty; therefore, it was not possible to refer to other studies in literature to perform a sample size calculation.

## Results

Of the 200 patients enrolled, 11 (5 from the control group and 6 from the experimental group) were excluded—4 due to intra- or post-operative complications and 7 due to the lack of more than one data item (e.g., subject not available, lack of cooperation, transfer to physiotherapy centre other than the one referred) (Table 1).

### Patient questionnaires

The following results were obtained:

HADS (Table 4): 12 out of 94 patients in the experimental group (12.8 %) manifested a state of anxiety, compared to 75 out of 95 in the control group (78.9 %) (*p* < 0.0001). Similarly, a state of depression was observed in 12 out of 94 patients in the experimental group (12.8 %) and in 70 out of 95 (73.7 %) in the control group (*p* < 0.0001). The differences between the experimental group and the control group were also significant within both the hip population and the knee population.

**Table 4:** Hospital anxiety and depression scale (HADS) results

	EXP	CTR	*p* value
Anxiety			
All patients	12/94 (12.8 %)	75/95 (78.9 %)	<0.0001*^,a^
Hip arthroplasty group	7/61 (11.5 %)	49/63 (77.8 %)	<0.0001*^,b^
Knee arthroplasty group	5/33 (15.2 %)	26/32 (81.3 %)	<0.0001*^,b^
Depression			
All patients	12/94 (12.8 %)	70/95 (73.7 %)	<0.0001*^,a^
Hip arthroplasty group	8/61 (13.1 %)	49/63 (77.8 %)	<0.0001*^,b^
Knee arthroplasty group	4/33 (12.1 %)	21/32 (65.6 %)	<0.0001*^,b^

Calculation performed on 95 patients in the EXP group (63 hips; 32 knees) and 94 patients in the CTR group (61 hips; 33 knees)

* Significance at *p* < 0.05

^a^Chi-squared test with Yate’s correction

^b^Fisher’s exact test

SF-36 (Table 5): With regard to the joint population (hip+knee), considerably higher average values were obtained in all 8 sub-scales in the experimental group compared to the control group in the pre-operative stage and in the two subsequent follow-ups. Furthermore, in the case of the ‘hip population’, the differences were significant in all subscales and follow-ups apart from the ‘role-physical’ sub-scale at the follow-up on day 45. For the ‘knee population’ the differences between the two groups only reached statistical significance in some of the sub-scales—all 4 sub-scales of the ‘Mental Component Scale’ both in the follow-up on day 45 and at 4 months, and the ‘Physical Functioning’ and the ‘General Health’ sub-scales on day 45.

**Table 5:** Results of the SF-36 questionnaire

Scale	Population (“pop”)	Pre-operative				45 days			
		No. of patients	EXP	CTR	*p* value	No. of patients	EXP	CTR	*p* value
*Sub-scales*									
Physical functioning	Joint pop	94 vs 95	45.5 ± 27.5	36.6 ± 21.3	0.0059*	94 vs 95	65.0 ± 21.2	48.2 ± 24.7	<0.0001*
	Hip pop	61 vs 63	48.2 ± 26.8	36.9 ± 21.7	0.0111*	61 vs 63	68.4 ± 20.5	49.7 ± 25.0	<0.0001*
	Knee pop	33 vs 32	40.5 ± 28.3	36.1 ± 20.9	0.4837	33 vs 32	58.6 ± 21.4	45.2 ± 24.2	0.0203*
Role-physical	Joint pop	94 vs 95	22.6 ± 30.2	13.9 ± 28.4	0.0439*	94 vs 95	18.6 ± 34.6	9.5 ± 23.1	0.0343*
	Hip pop	61 vs 63	23.0 ± 30.0	11.9 ± 24.9	0.0281*	61 vs 63	19.7 ± 36.0	9.1 ± 23.5	0.0568
	Knee pop	33 vs 32	22.0 ± 31.1	18.0 ± 34.3	0.6239	33 vs 32	16.7 ± 32.3	10.2 ± 22.8	0.3523
Bodily pain	Joint pop	94 vs 95	40.1 ± 19.3	30.5 ± 18.3	0.0005*	94 vs 95	70.5 ± 23.6	58.6 ± 25.6	0.0011*
	Hip pop	61 vs 63	43.3 ± 21.2	30.5 ± 19.1	0.0006*	61 vs 63	77.0 ± 21.2	63.0 ± 24.8	0.001*
	Knee pop	33 vs 32	34.2 ± 13.7	30.4 ± 17.0	0.3283	33 vs 32	58.5 ± 23.6	49.9 ± 25.3	0.1588
General health	Joint pop	94 vs 95	66.8 ± 18.0	57.4 ± 20.1	0.0008*	94 vs 95	79.4 ± 19.0	66.0 ± 22.4	<0.0001*
	Hip pop	61 vs 63	69.8 ± 16.4	56.0 ± 22.1	0.0001*	61 vs 63	80.7 ± 19.9	66.1 ± 24.0	0.0003*
	Knee pop	33 vs 32	61.4 ± 19.7	60.2 ± 15.3	0.7787	33 vs 32	77.1 ± 17.2	65.8 ± 18.9	0.0142*
Vitality	Joint pop	94 vs 95	66.8 ± 18.0	41.1 ± 19.8	0.0001*	94 vs 95	71.9 ± 19.9	37.3 ± 22.6	<0.0001*
	Hip pop	61 vs 63	53.4 ± 16.1	38.6 ± 21.0	<0.0001*	61 vs 63	74.8 ± 18.0	37.9 ± 23.6	<0.0001*
	Knee pop	33 vs 32	50.3 ± 23.5	46.1 ± 16.3	0.4030	33 vs 32	66.4 ± 22.2	36.3 ± 20.8	<0.0001*
Social functioning	Joint pop	94 vs 95	63.6 ± 23.9	53.2 ± 27.9	0.0065*	94 vs 95	77.5 ± 23.2	45.3 ± 26.9	<0.0001*
	Hip pop	61 vs 63	64.5 ± 23.4	49.4 ± 28.3	0.0015*	61 vs 63	80.1 ± 21.3	45.9 ± 29.1	<0.0001*
	Knee pop	33 vs 32	61.7 ± 25.2	60.5 ± 25.8	0.8507	33 vs 32	72.7 ± 25.6	44.1 ± 22.2	<0.0001*
Role-emotional	Joint pop	94 vs 95	48.6 ± 39.9	35.8 ± 38.4	0.0258*	94 vs 95	79.8 ± 33.6	28.4 ± 35.7	<0.0001*
	Hip pop	61 vs 63	50.8 ± 41.1	33.3 ± 37.4	0.0147*	61 vs 63	84.7 ± 29.5	29.1 ± 37.1	<0.0001*
	Knee pop	33 vs 32	44.4 ± 37.9	40.6 ± 40.4	0.6955	33 vs 32	70.7 ± 38.9	27.1 ± 33.3	<0.0001*
Mental health	Joint pop	94 vs 95	67.1 ± 19.4	54.3 ± 22.6	<0.0001*	94 vs 95	82.3 ± 20.4	46.0 ± 26.9	<0.0001*
	Hip pop	61 vs 63	67.9 ± 18.5	51.0 ± 22.4	<0.0001*	61 vs 63	83.3 ± 19.4	47.7 ± 28.4	<0.0001*
	Knee pop	33 vs 32	65.7 ± 21.2	60.8 ± 21.8	0.3568	33 vs 32	80.6 ± 22.3	42.8 ± 23.9	<0.0001*
*Physical component and mental component summaries*									
Physical component summary	Joint pop	94 vs 95	33.8 ± 9.3	31.3 ± 7.4	0.0466*	94 vs 95	39.2 ± 8.4	39.2 ± 7.8	0.9665
	Hip pop	61 vs 63	35.1 ± 9.1	31.5 ± 8.1	0.0192*	61 vs 63	40.5 ± 8.9	39.8 ± 8.0	0.6368
	Knee pop	33 vs 32	31.2 ± 9.4	31.0 ± 5.8	0.9075	33 vs 32	36.8 ± 6.9	37.9 ± 7.5	0.5397
Mental component summary	Joint pop	94 vs 95	47.8 ± 11.3	41.8 ± 12.2	0.0005*	94 vs 95	56.7 ± 11.6	35.0 ± 13.0	<0.0001*
	Hip pop	61 vs 63	48.1 ± 11.1	39.9 ± 11.9	0.0001*	61 vs 63	57.6 ± 10.6	35.4 ± 14.1	<0.0001*
	Knee pop	33 vs 32	47.4 ± 11.7	45.5 ± 12.0	0.5291	33 vs 32	55.0 ± 13.3	34.4 ± 10.9	<0.0001*

Scale	Population (“pop”)	4 months				ANOVA			
		No. of patients	EXP	CTR	*p* value	No. of patients	Main effect of treatment	Main effect of follow-up	Interaction between treatment allocation and follow-up
*Sub-scales*									
Physical functioning	Joint pop	93 vs 94	86.6 ± 14.3	74.3 ± 25.5	<0.0001*	93 vs 94	<0.0001*	<0.0001*	0.1125
	Hip pop	60 vs 63	88.4 ± 13.8	71.8 ± 27.8	<0.0001*	60 vs 63	<0.0001*	<0.0001*	0.2807
	Knee pop	33 vs 31	83.3 ± 14.7	79.4 ± 19.7	0.3700	33 vs 31	0.0490*	<0.0001*	0.2449
Role-physical	Joint pop	93 vs 94	79.3 ± 36.4	66.3 ± 45.2	0.0319*	93 vs 94	0.0014*	<0.0001*	0.7794
	Hip pop	60 vs 63	84.6 ± 35.1	62.9 ± 46.4	0.0040*	60 vs 63	0.0003*	<0.0001*	0.3100
	Knee pop	33 vs 31	69.7 ± 37.4	73.4 ± 42.3	0.7124	33 vs 31	0.7136	<0.0001*	0.6738
Bodily pain	Joint pop	93 vs 94	76.7 ± 23.2	68.6 ± 28.0	0.0326*	93 vs 94	<0.0001*	<0.0001*	0.6229
	Hip pop	60 vs 63	80.4 ± 23.5	69.0 ± 28.8	0.0181*	60 vs 63	0.0001*	<0.0001*	0.8813
	Knee pop	33 vs 31	70.1 ± 21.5	67.8 ± 26.8	0.7148	33 vs 31	0.1317	<0.0001*	0.5534
General health	Joint pop	93 vs 94	78.9 ± 19.7	67.7 ± 25.8	0.0011*	93 vs 94	<0.0001*	<0.0001*	0.4014
	Hip pop	60 vs 63	81.1 ± 18.9	64.4 ± 27.5	0.0001*	60 vs 63	<0.0001*	<0.0001*	0.6899
	Knee pop	33 vs 31	74.9 ± 20.7	74.4 ± 21.1	0.9158	33 vs 31	0.1991	<0.0001*	0.0546
Vitality	Joint pop	93 vs 94	74.9 ± 20.7	74.4 ± 21.1	<0.0001*	93 vs 94	<0.0001*	<0.0001*	<0.0001*
	Hip pop	60 vs 63	61.3 ± 23.3	40.1 ± 22.6	<0.0001*	60 vs 63	<0.0001*	<0.0001*	<0.0001*
	Knee pop	33 vs 31	53.8 ± 24.4	39.8 ± 19.8	0.0150*	33 vs 31	0.0001*	0.3827	0.0002*
Social functioning	Joint pop	93 vs 94	75.7 ± 23.5	56.0 ± 23.8	<0.0001*	93 vs 94	<0.0001*	0.0009*	<0.0001*
	Hip pop	60 vs 63	76.9 ± 21.8	56.2 ± 24.7	<0.0001*	60 vs 63	<0.0001*	0.0005*	0.0005*
	Knee pop	33 vs 31	73.5 ± 26.5	55.6 ± 22.3	0.0051*	33 vs 31	0.0006*	0.2377	0.0018*
Role-emotional	Joint pop	93 vs 94	78.9 ± 33.6	60.3 ± 39.2	0.0006*	93 vs 94	<0.0001*	<0.0001*	<0.0001*
	Hip pop	60 vs 63	81.1 ± 32.1	63.5 ± 40.0	0.0080*	60 vs 63	<0.0001*	<0.0001*	<0.0001*
	Knee pop	33 vs 31	74.8 ± 36.4	53.8 ± 37.2	0.0259*	33 vs 31	0.0005*	0.0031*	0.0065*
Mental health	Joint pop	93 vs 94	67.8 ± 26.6	50.1 ± 26.2	<0.0001*	93 vs 94	<0.0001*	0.0130*	<0.0001*
	Hip pop	60 vs 63	68.7 ± 27.5	50.0 ± 27.8	0.0003*	60 vs 63	<0.0001*	0.0040*	<0.0001*
	Knee pop	33 vs 31	66.3 ± 25.0	50.3 ± 23.1	0.0101*	33 vs 31	<0.0001*	0.4050	<0.0001*
*Physical component and mental component summaries*									
Physical component summary	Joint pop	93 vs 94	52.2 ± 7.5	48.8 ± 10.9	0.0135*	93 vs 94	0.0310*	<0.0001*	0.0850
	Hip pop	60 vs 63	53.7 ± 7.6	47.8 ± 11.4	0.0009*	60 vs 63	0.0048*	<0.0001*	0.0201*
	Knee pop	33 vs 31	49.5 ± 6.6	50.9 ± 9.8	0.5114	33 vs 31	0.5968	<0.0001*	0.8457
Mental component summary	Joint pop	93 vs 94	46.6 ± 13.0	37.1 ± 13.6	<0.0001*	93 vs 94	<0.0001*	0.0002*	<0.0001*
	Hip pop	60 vs 63	47.1 ± 13.1	38.2 ± 14.4	0.0004*	60 vs 63	<0.0001*	0.0053*	<0.0001*
	Knee pop	33 vs 31	45.5 ± 13.1	35.1 ± 11.6	0.0013*	33 vs 31	<0.0001*	0.0024*	<0.0001*

* Significance at *p* < 0.05

**Fig. 1:**
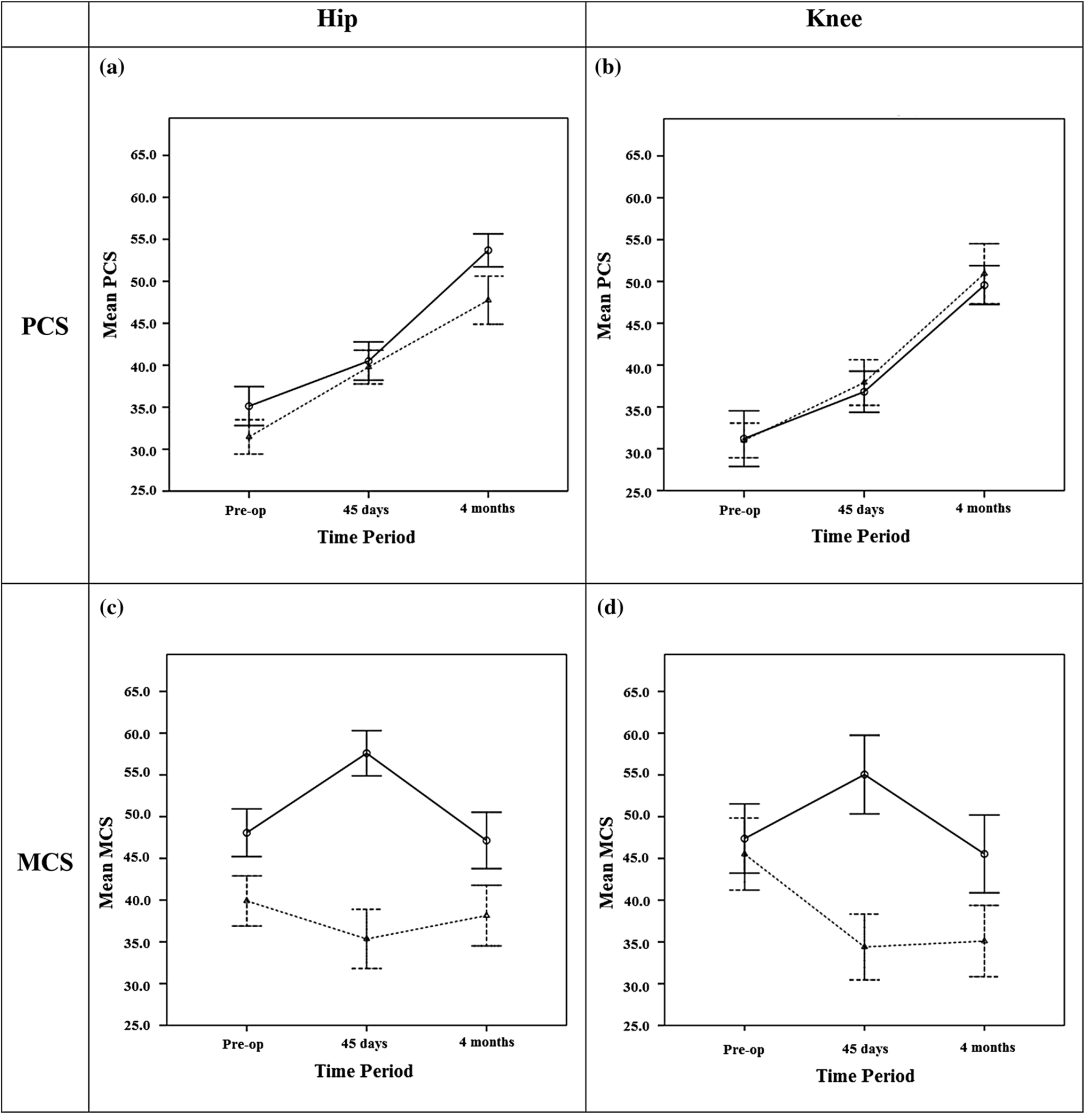
Temporal trend of the ‘Physical Component Scale’ (PCS) and the ‘Mental Component Scale’ (MSC) of the SF-36 questionnaire for the hip population ((**a**) and (**c**), respectively) and for the knee population ((**b**) and (**d**), respectively). The *solid lines* indicate the experimental group while the *dashed lines* indicate the control group. Means are shown as *circles* while the *bars* represent the 95 % confidence interval. An evident overlapping between the *bars* indicates lack of significant statistical difference

With regard to the ‘Physical Component Scale’, a similar temporal trend of values was observed in the two study groups, significantly increasing over time in both groups, taking into consideration both the joint population and the two populations (hip and knee) separately (*p* < 0.0001). For the joint population, the values were significantly higher as a whole in the experimental group compared to the control group (*p* = 0.0310) and, in particular, were higher in the pre-operative visit (*p* = 0.0466) and in the follow-up at 4 months (*p* = 0.0135), while there was no significant difference in the follow-up on day 45. The same consideration applies for the ‘hip population’ (Fig. 1a), while there was no significant difference between the groups in the ‘knee population’ (Fig. 1b) at any follow-up.

With regard to the ‘Mental Component Scale’, in both the joint population and the two hip and knee populations separately, an exact opposite temporal trend was observed in the experimental group compared to the control group (*p* < 0.0001), with generally higher scores in the experimental group (*p* < 0.0001). The differences are significant in the pre-operative stage, on day 45 and at 4 months after the operation in both the joint population (*p* = 0.0005, *p* < 0.0001, *p* < 0.0001, respectively) and in the ‘hip population’ (*p* = 0.0001, *p* < 0.001, *p* = 0.0004, respectively) (Fig. 1c). In the ‘knee population’, a significant difference was observed on day 45 (*p* < 0.0001) and at 4 months (*p* = 0.0013) but not at the pre-operative visit (Fig. 1d).

The average values and the statistical significances are stated in Table 5.

### Physiotherapy sheet

The following results were obtained:

**‘**Delta autonomy days’ (Table 6): with regard to the ‘hip population’, a significant difference between the experimental group and the control group was observed, with the physiotherapy objective being reached, on average, after 6.7 ± 1.8 days (range 4–12) and 7.9 ± 2.2 days (range 0–13), respectively, after the operation (*p* = 0.0015). The difference between the experimental group and the control group in the ‘knee population’ did not reach the statistical significance [8.1 ± 2.4 days (range 5–16) vs 8.8 ± 2.3 days (range 5–14)].

**Table 6:** Physiotherapy results

		EXP	CTR	*p* value
Delta autonomy days [mean ± SD (days)]	All patients	7.2 ± 2.2	8.2 ± 2.3	0.0023*
	Hip arthroplasty group	6.7 ± 1.8	7.9 ± 2.2	0.0015*
	Knee arthroplasty group	8.1 ± 2.4	8.8 ± 2.3	0.2424

Calculation performed on 92 patients in the EXP group (59 hips; 33 knees) and 93 patients in the CTR group (61 hips; 32 knees). Data analysed by Student’s *t* test and confirmed by Mann–Whitney test. *p*-values refer to *t* test

* Significance at *p* < 0.05

## Discussion

The study highlighted that the group that received psychological support presented a significantly lower number of patients with a state of anxiety and depression upon discharge compared to the control group.

With regard to the ‘Physical Component Scale’ of the SF-36 score, an improvement in scores over time was observed in both the experimental group and the control group, although with generally higher scores in the experimental group. As regards the population with hip arthroplasty, the scores were significantly higher in the experimental group in the pre-operative stage (after the first session with the psychologist) and in the follow-up at 4 months. In the population with knee arthroplasty, a significant difference between the two groups was not observed in any of the follow-ups. This difference in the results between the patients with hip operations and those with knee operations could be due to the fact that in the case of knee arthroplasty the physical component (also understood as physical pain and the role it plays in the perceived quality of health) has more prominence and may be less influenced by psychological support.

With regard to the ‘Mental Component Scale’ of the SF-36 score, the results of the overall population (hip+knee) were significantly better in the subjects provided with psychological support in the pre-operative stage and in the two subsequent follow-ups. These values were already higher after the first session with the psychologist, taking into consideration the two populations separately (hip and knee), with significant differences in all cases, apart from the pre-operative stage for the patients undergoing knee operations. In our opinion, these results indicate that the psychological support provided during admission, the hospital stay and rehabilitation led to an improvement in mental well-being in both the short and long term. In addition, the fact that the score in patients who received psychological support increased at the follow-up on day 45 and then decreased at 4 months (but remained higher than the control group) shows, in our opinion, the effectiveness and the impact of psychological therapy, especially in the initial period after the surgery up to the evaluation on day 45. Afterwards, the improvement achieved would build up even more over time from a physical and, consequently, emotional point of view.

Lastly, it was observed that the patients provided with psychological support who underwent hip arthroplasty reached the physiotherapy objective (i.e., the patient ability to walk 50 metres independently and to climb 10 steps) 1.2 days earlier, on average, compared to the patients who did not receive this therapy (*p* = 0.0015). This improvement was also apparent in the population with knee arthroplasty, although the difference between the study and the control group was in this case not significant. In our opinion, the incorporation of psychological support in the clinical, surgical and rehabilitation procedure could therefore also be an economic innovation. In fact, in addition to determining an improvement in the psycho-physical well-being of the patient, it could bring about a reduction in costs of patient treatment as a consequence of the reduction in rehabilitation time at the rehabilitation centre (currently, in the case of our facility, set at 8 days following the 5-day post-operative stay in hospital). Considering the outcome obtained in this study and given that, in the case of this rehabilitation centre, the cost of the stay amounts to EUR 175 per day for each patient (current cost as of 2014), early discharge by 1 day compared to the current standard would correspond to a saving in rehabilitation costs of EUR 175 gross per patient. This saving should be compared with the gross cost per patient for psychological support, which is calculated at EUR 63 gross (taking into consideration a gross cost of EUR 31.50 per hour and considering that each patient participated in four sessions, each lasting approximately half an hour). Making the calculation with approximately 600 patients who undergo primary hip replacement each year at our facility, the gross total annual saving would amount to EUR 67,200.

In summary, in the patients who received psychological support, a lower incidence of anxiety and depression and better mental well-being was observed compared to the patients who did not receive this therapy. In the patients who underwent hip arthroplasty, a reduction of an average of 1.2 days in the period to reach the physiotherapy objective was observed in the group that received psychological support compared to the control group.

This study is significant because, to the best of our knowledge, it is the first controlled study in this therapeutic field. It would be interesting to design a study focused on patients with more complex diagnoses (for example patients undergoing revision surgery), or by comparing protocols with a different number of psychological support visits to determine which protocol could be the most cost-effective.
